# Editor's Note to ‘Identification of peptide inhibitors of pre-mRNA splicing derived from the essential interaction domains of CDC5L and PLRG1’

**DOI:** 10.1093/nar/gkac623

**Published:** 2022-07-15

*Nucleic Acids Research*, Volume 31, Issue 21, 1 November 2003, Pages 6104–6116, https://doi.org/10.1093/nar/gkg817

Following allegations in 2021 of image manipulation, the authors’ institution analysed the article and confirmed that lanes 7–8 and 9–10 in Figure 6 are duplicates. The institution notified the journal of this duplication in March 2022, and the authors have unreservedly apologised for this error. While the lanes show a negative result and the Editors believe the duplication to be a genuine mistake, they cannot confirm whether or not the results and conclusions presented in the article are affected because the original data are no longer available. The Editors, therefore, advise readers to examine the details of this study with care.

Julian E. Sale and Barry L. Stoddard

Senior Executive Editors

